# Lab-Made Electrochemical System with Flexible rGO/PAni Electrode for Selective Multiclass Pharmaceutical Detection

**DOI:** 10.1021/acsomega.5c11695

**Published:** 2026-01-21

**Authors:** Layne Taynara Santos Zanon, Vitor Hugo Neto Martins, Liriana Mara Roveda, Luis Gustavo do Espírito Santo Mendes, Claudio Teodoro de Carvalho, Raphael Rodrigues, Victor Hugo Rodrigues de Souza, Magno Aparecido Gonçalves Trindade

**Affiliations:** Faculdade de Ciências Exatas e Tecnologia, Universidade Federal da Grande Dourados, Rodovia Dourados-Itahum, km 12, Dourados, MS 79804-970, Brazil

## Abstract

The development of efficient analytical procedures for environmental applications increasingly relies on electrochemical techniques and their associated systems, which are prized for their high sensitivity, moderate cost, and portability. To overcome the limitations of conventional electrochemical setups, this study introduces an alternative lab-made electrochemical cell design incorporating a flexible reduced graphene oxide/polyaniline (rGO/PAni) composite electrode. The free-standing nanocomposite electrodes based on rGO/PAni, characterized by their high electrical conductivity, thermal stability, and large surface area, were strategically chosen to enhance the electrode performance. This thick, malleable, and easy-to-handle film provides a satisfactory fit with an alternative lab-made electrochemical cell. As a proof-of-concept, this system was successfully applied to the simultaneous detection of multiclass pharmaceutical contaminants—acetaminophen, salicylic acid, and norfloxacin—recognized as emerging environmental pollutants in groundwater samples. The adaptable nature and advantageous properties of the rGO/PAni-based working electrode, coupled with the optimized lab-made cell configuration, demonstrate the potential of this alternative electrochemical system for selective environmental electroanalysis.

## Graphical abstract

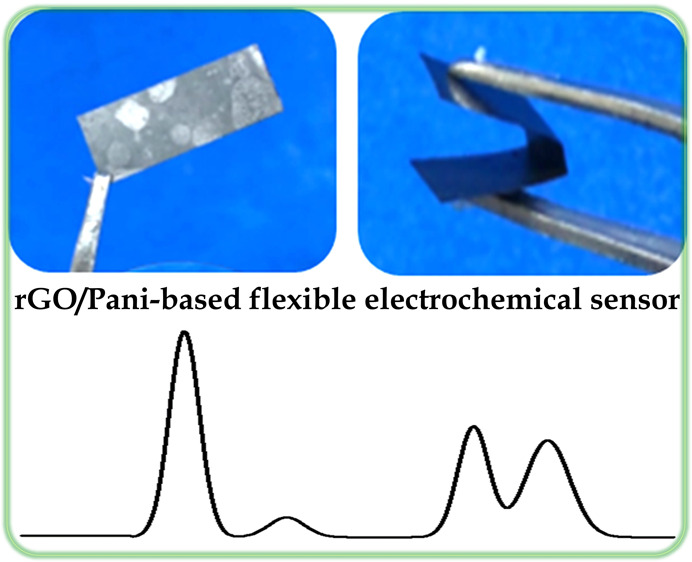

## 1 Introduction

Electrochemical systems are well-established analytical tools for environmental monitoring, praised for their inherent high-sensitivity approaches, cost-effectiveness, user-friendliness, and potential for portable applications.^1–4^ Despite advancements in analytical performance offered by modern electrochemical system designs, their practical complexities (e.g., automated workflows^5^ and high-performance and flexible sensors) often hinder widespread adoption, particularly in laboratories with limited resources or specialized expertise to manage such sophisticated setups. Recent achievements have led to the development of low-cost alternative electrochemical setups.^6–9^ However, a persistent challenge remains in further enhancing these alternative systems and the use of sustainable or reusable materials for building modified electrochemical cells and their associated components.

Typically, these cells feature individual electrodes, which are suspended from the top, often secured within a cap.^8–10^ A significant challenge with these traditional three-electrode electrochemical setups lies in their susceptibility to displacement during essential operations such as solution stirring, which is crucial for ensuring adequate mass transport to the electrode surface and/or maintaining a clean working area.^6–9^ Additionally, such movement can lead to the formation of bubbles on the working electrode (WE) surface, which reduces its available electrochemically active surface area (ECSA) and can negatively impact the measured signals.^8,9,11,12^ Critically, random stirring of the supporting electrolyte can alter the interelectrode distance between the WE and the reference electrode (RE). This dynamic distance introduces an uncontrolled and variable potential drop across both electrodes, further distorting the measured electrochemical response.^13^ For laboratories with infrastructural limitations, these simple issues can challenge the practical development and reliable operation of electrochemical devices. To overcome these obstacles and ensure consistent experimental results, we have developed innovative electrochemical cell designs that offer improved stability and operational reliability, enabling researchers to conduct effective experiments despite infrastructure limitations.^6–9^

Recognizing these limitations, our recent electroanalytical research has focused on developing modified electrochemical systems with enhanced performance characteristics.^6–9^ This involves the optimization of the electrode configurations tailored to strategically relocate the WE to the bottom section of the electrochemical cell while maintaining the auxiliary and reference electrodes at the top.^6,7,9^ This simple rearrangement offered several important advantages, especially since it has undergone several modifications that have evolved from its application in liquid–liquid microextraction coupled to electroanalysis (in a single device) to the current emphasis on the study of new electrode materials for use as WE.^6–9^ This design necessitates that the WE material exhibits properties such as reduced susceptibility to contaminant adsorption from the sample and a broad working potential range, among other characteristics desirable for a multifunctional electrochemical system. Importantly, fixing the WE at the bottom of the cell enables efficient mechanical stirring of the supporting electrolyte solution, which serves to effectively renew its surface by minimizing and/or eliminating the adsorption of electrogenerated products.^6–9^

While boron-doped diamond (BDD) is a suitable material for electrochemical applications,^7^ the modified electrochemical cell designs may pose challenges for fragile materials like BDD due to adaptation difficulties. The bottom-mounted WE configuration is particularly well-suited for malleable, flexible, and easily handled electrode films, ensuring a secure and consistent electrode–electrolyte interface. For example, composite materials such as reduced graphene oxide and polyaniline (rGO/PAni)^14,15^ offer large surface areas and unique electronic properties, leading to enhanced sensitivity and broader applicability in electrochemical sensing. The integration of such tailored materials with optimized cell designs holds significant promise for overcoming the limitations of traditional electrode materials and cell configurations.

The rGO/PAni electrode incorporates graphene, which possesses a multitude of properties, such as high electrical conductivity, surface area, and thermal and mechanical stability, especially in its reduced form.^14,15^ This material, processed into independent thin films, enables the fabrication of thin, light, malleable, and flexible electrodes easily integrated into various devices.^14,15^ Addressing existing challenges, this research introduces a lab-made electrochemical cell using readily available and reusable materials. The configuration has an alternative lab-made stirring system that enables efficient electrode surface renewal, minimizing interference from adsorbed electrogenerated products. In addition, the bottom-mounted WE design, enabled by the rGO/PAni film’s flexibility, prevents electrode displacement during mechanical stirring, ensuring stable and reproducible measurements.

The proof-of-concept application in groundwater showed effective selective detection of multiclass pharmaceuticals (acetaminophen, salicylic acid, and norfloxacin, named as emerging environmental concerns^16,17^ ), highlighting its potential for electroanalysis of organic contaminants. Namely, several studies have reported on the detection of these pharmaceuticals, either individually or in combination, using various electrode materials and cell configurations.^18–22^ However, no studies have reported the use of free-standing nanocomposite electrodes based on rGO and PAni, produced by a doctor blade, for the simultaneous detection of target analytes. Given the critical and increasing threat of pharmaceutical water pollution to the environment and human health, developing such efficient monitoring tools is essential for informed assessment and mitigation efforts.

## 2 Experimental Section

### 2.1 Synthesis of the rGO/PAni Electrode

#### 2.1.1 Synthesis of Graphene Oxide and Polyaniline

Graphene oxide (GO) was prepared using a modified Hummers’ method as previously developed by Martins and co-workers.^15^ Briefly, graphite flakes (Grafine, Nacional de Grafite LTDA) were preoxidized using potassium persulfate (K_2_S_2_O_8_, 99%, Sigma-Aldrich)/phosphorus pentoxide (P_2_O_5_, 99%, Sigma-Aldrich) in sulfuric acid (H_2_SO_4_, 96%, Sigma-Aldrich) at 80 °C. The resulting material was then fully oxidized by potassium permanganate (KMnO_4_, 99%, Sigma-Aldrich) in sulfuric acid at 0 °C followed by the addition of hydrogen peroxide (30%, v/v, Sigma-Aldrich), which led to the dispersion developing a bright yellow coloration. Subsequently, the GO was rinsed with a 3.4% hydrochloric acid solution, filtered, additionally washed with acetone, and finally air-dried.

Polyaniline was prepared using a rapid mixing method, modifying a previously published protocol.^23^ In this context, 50 mL of a 1.0 mol L^–1^ hydrochloric acid solution with 16.0 mmol of aniline (99%, Sigma-Aldrich) was mixed with 50 mL of the same doping acid containing 4.0 mmol of ammonium persulfate ((NH_4_)_2_S_2_O_8_, 98%, Sigma-Aldrich). This solution was stirred immediately after mixing. The PAni (green emeraldine salt) was centrifuged and neutralized to the emeraldine base form by washing it with an ammonium hydroxide solution (10%) followed by water until a neutral medium (pH ≅ 7.0) was achieved.

#### 2.1.2 Synthesis of Thin Films

The rGO/PAni films were prepared from the mixture of GO and PAni in the emeraldine base form according to the methodology previously reported by Martins and co-workers.^15^ Briefly, a GO dispersion was prepared by mechanically stirring (vortexing) a mixture of 100 mg (2.0%) of powdered GO in 2.5 mL of deionized water. PAni (blue emeraldine base) (12.5 mg, 0.25%) was dispersed in another flask by adding 2.5 mL of deionized water and sonicated for 10 min in an ultrasonic bath. These percentages of GO (2.0%) and PAni (0.25%) were based on the total mass of water (5.0 g) used to disperse the precursors. Both dispersions (GO and PAni) were mixed and then mechanically stirred by using a vortex until a homogeneous green viscous mixture was obtained. The final material (gel) was uniformly coated onto glass substrates using a doctor’s blade technique. The film was dried at 60 °C for 30 min, followed by 12 h chemical reduction using hydrazine vapor, resulting in an rGO/PAni freestanding electrode.

### 2.2 Instrumentation, Electrochemical Cell Design, and Experimental Setup

A reverse osmosis water purifier (Gehaka, model OS 10 LTXE) was utilized to obtain deionized water (*R* ≥ 18.2 MΩ·cm) for the preparation of all working solutions. The pH measurements were performed in a combined glass electrode (Hanna, model HI 1131B) connected to a digital pH meter (Hanna, model HI 3221). Voltammetric measurements and experimental control were conducted using a potentiostat/galvanostat (Metrohm, model PGSTAT 204) in conjunction with the NOVA 2.1.6 Software. The crystalline structure of the rGO/PAni electrode was determined using a Shimadzu XRD-6000 instrument with Cu Kα radiation (λ = 1.5418 Å) and an incident angle of 0.1°. A SEM-FEG (Tescan, Mira-3) was used for morphology investigation at an accelerating voltage of 10.0 kV and a working distance of 5 mm. A SEM (Phenom-World, PRO-X) was used for the cross-sectional micrographs at an accelerating voltage of 10.0 kV and working distance of 5 mm. Sheet resistance measurements were performed using a Four-Point Probe (Ossila Ltd.) in conjunction with the Ossila Sheet Resistance Lite software. Forty points were analyzed in portions of the film with a rectangular format of 200 × 90 mm.

The lab-made electrochemical cell (120 mL maximum capacity) was constructed from machined acrylic material and comprises a primary cylindrical reservoir with a detachable lid. This custom-made device was designed for electrode integration at the base and mechanical stirring apparatus placement at the top. As illustrated in Figure 1A,B, threaded rods secure both the lower and upper sections. The lower section was specifically designed to hold the rGO/PAni working electrode (WE), while the upper section features a circular form to fit the lid, which contains holes for the reference electrode (RE) and auxiliary electrode (AE) as well as the stirring rod. Threaded rods with screws facilitate straightforward assembly and disassembly during WE electrode replacement. In the assembled device illustration, the centrally positioned stirring rod is operated by a printer motor. Figure 1C provides an overview of all cell components, emphasizing the convergent base design that promotes the efficient delivery of analyte to the WE surface.

**Figure 1:**
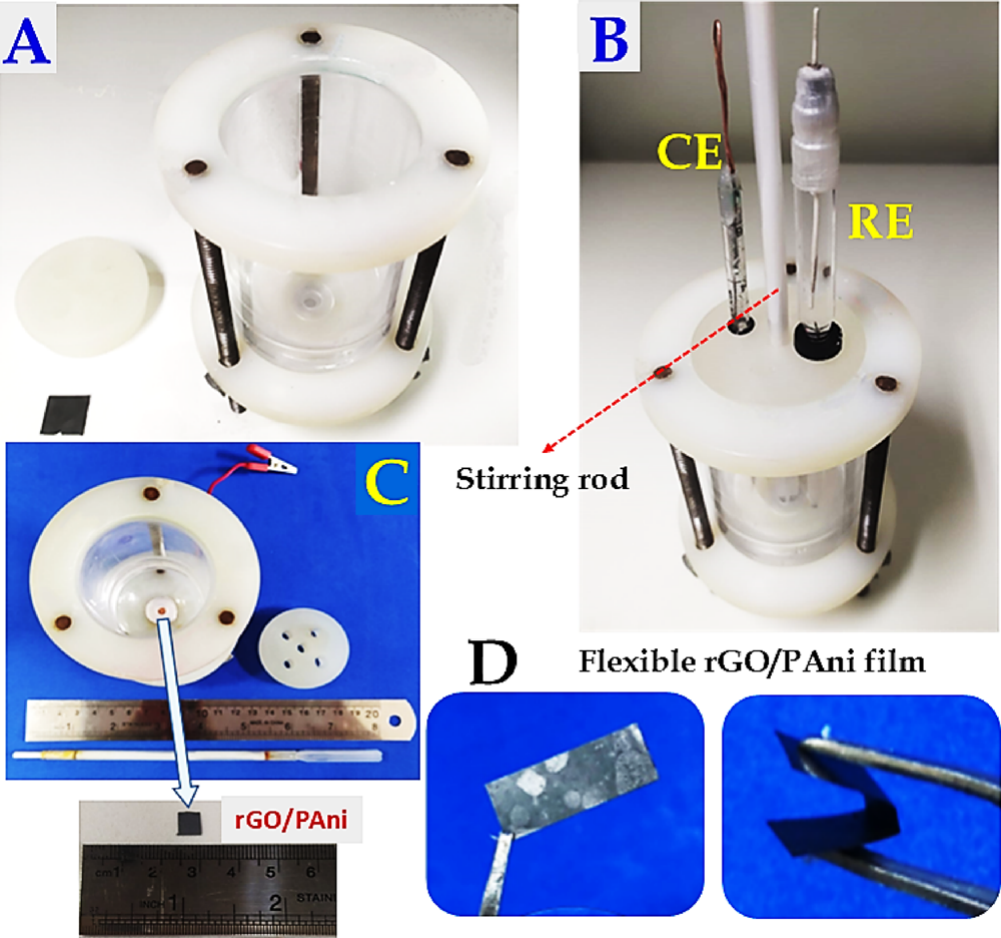
Images showing the components of the electrochemical system: (A) the lab-made electrochemical cell, (B) partially assembled electrochemical cell with the auxiliary (Pt plate) and the reference (Ag|AgCl|KCl_sat._) electrodes and the lab-made mechanical stirring system on the center of the cell cover, (C) top view of the electrochemical cell holding a 10 × 10 mm rGO/PAni plate as working electrode, and (D) image showing the flexibility of the rGO/PAni film.

A key feature is the bottom-mounted working electrode, a 10 × 10 mm rGO/PAni plate (Figure 1A,D). To enhance the system’s functionality, we also integrated a lab-made mechanical stirring rod (Figure 1B). This rod is powered by a DC Motor RS550 (12 V, 30,000 rpm, 35 × 80 mm) specifically adapted to connect to the top of the cell. The stirring mechanism itself comprises a cylinder with a helix to ensure efficient solution mixing. We controlled the stirring speed using an adjustable AC/DC power supply (1.5–12 V output voltage), which enabled precise control over the stirring operation. This integrated stirring system was essential for cleaning the WE surface by removing adsorbed electrogenerated products or sample interferents, reactivating the electrochemical activity of the electrode. All components of this apparatus were lab-made using readily available, low-cost, reusable, and/or disposable materials.

### 2.3 Voltammetric and Chromatographic Studies

The voltammetric measurements were performed on a target spiked solution (within an appropriate electrolytic medium). Unless otherwise indicated, all measurements were conducted in triplicate, and standard deviations were calculated. After each electrochemical measurement, the rGO/PAni electrode surface was cleaned by mechanically stirring the solution, which applied a turbulent flow directly to the WE surface. After the original voltammograms were registered, signal transformation was performed using the baseline-corrected procedure we highlighted in a previous work.

For high-performance liquid chromatography with diode-array detection (HPLC-DAD), a Zorbax Eclipse Plus C18 column (Agilent Technologies, 250 × 4.6 mm, 5 μm) was employed, with the column temperature controlled at 25 °C. The mobile phase comprised methanol and water acidified with 0.10% acetic acid, and the detection of target analytes was set at 240 nm. A 25 μL injection volume was used. Prior to injection, all standards and samples were filtered through a 0.25 μm nylon filter (Millipore) to ensure the sample purity. The gradient elution program started with 60% methanol at a flow rate of 0.75 mL min^–1^ for the first 4 min. Subsequently, the flow rate was increased to 1.25 mL min^–1^, maintained until 6 min, and then re-equilibrated to the starting conditions.

## 3 Results and Discussion

### 3.1 Characterization of the rGO/PAni

The structural arrangement of rGO/PAni was investigated through X-ray diffractometry (XRD), and the result is depicted in Figure S1. The XRD pattern of the composite displays a broad diffraction peak centered at 2θ = 22.8°, which corresponds to the (002) reflection plane of the rGO, and its broadness reveals limited ordering of the sheets throughout the stacking direction.^15,24^ Moreover, peaks at 2θ = 19.0 and 25.1° can be assigned to the (100) and (110) planes of the partially crystalline structure of PAni, consistent with its emeraldine salt form in a pseudoorthorhombic unit cell. The broad peak corresponding to rGO covers all peaks related to the PAni because of the π–π interaction between the carbon nanostructure and the phenyl rings of the polymer backbone.^25–27^

The morphology of the composite was observed by using scanning electron microscopy (SEM), and the micrographs are illustrated in Figure 2. The SEM image shown in Figure 2a exhibits a wrinkled structure profile, typically related to graphene sheets. The presence of the fibrillar structures of PAni covered by some graphene layers is observed in the higher-magnification SEM image in Figure 2b. Such a profile is a consequence of the experimental procedure adopted herein, in which the emeraldine base form of PAni turns into emeraldine salt when in contact with the GO, increasing the interaction between both materials, as described elsewhere.^15,28^ Furthermore, the cross-sectional micrograph in Figure 2c exhibits the stacking of graphene sheets and a porous structure resulting from the hydrazine vapor reduction adopted herein, which produces N_2_ gas as a coproduct. The film width, as measured from the cross-sectional micrograph, was approximately 45.0 ± 4.7 μm. The sheet resistance of the freestanding film, evaluated through four-point probe analysis, was about 62 ± 10 Ω cm^–1^. The low sheet resistance highlights the effectiveness of the chemical reduction of graphene oxide (GO) to reduced graphene oxide (rGO), resulting in rGO/PAni freestanding films that are suitable for use as conductive electrodes.^21,22^

**Figure 2:**
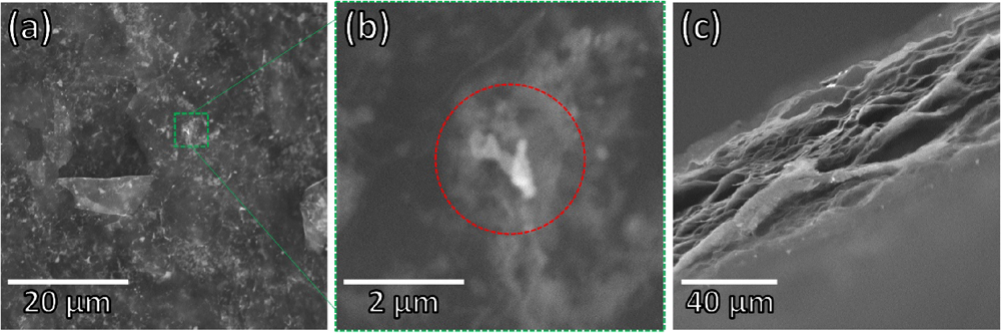
Scanning electron microscopy (SEM) images of rGO/PAni of the (a, b) surface and (c) cross-sectional region.

### 3.2 Cyclic Staircase Voltammetry Profile for the rGO/PAni Electrode

To evaluate the functionality of the lab-made electrochemical cell having the bottom-mounted rGO/PAni electrode, some important parameters of staircase cyclic voltammetry were studied using potassium ferrocyanide/ferricyanide ([Fe­(CN)_6_]^4–^/[Fe­(CN)_6_]^3–^) as a redox probe. Initial tests performed at the rGO/PAni working electrode across a range of scan rates (10–500 mV s^–1^) revealed typical well-defined voltammetric profiles characteristic of the redox probe’s electrochemical reaction (Figure S2A, SM). As the scan rate increased, the peaks became sharper and larger; however, these peaks remained observable even at higher scan rates. Despite considerable capacitive current—resulting from the synergistic increase in the electrode’s active surface area—the observed voltammetric profiles for the redox probe on the rGO/PAni electrode composite indicate both a broad usable potential window and minimal charge transfer kinetic hindrance. Further evidence of proper electrode function is the peak-to-peak separation of approximately 100 mV at lower scan rates and an anodic-to-cathodic peak current ratio of nearly unity (Figure S2A, SM). While this specific configuration of an rGO/PAni-based electrode in an electrochemical device has not been previously reported, its characteristic voltammetric profile provides evidence of a properly functioning system. The observed reversible behavior indicates that our rGO/PAni electrode delivers electrochemical performance qualitatively consistent with previous studies on typical carbonaceous electrodes using the same redox probe.^22^

Since electron transfer appears to involve a freely diffusive process, we used the Randles–Sevcik equation (eq 1) to calculate the electrochemically active surface area (ECSA) for the proposed rGO/PAni working electrode. Considering the regression equation (Figure S2B), the calculated ECSA was 0.35 mm^2^, which is significantly larger than its geometric surface area (0.20 cm^2^). This clearly indicates the significant contribution of the conductive materials incorporated into the electrode’s construction to its overall electrochemical performance. Furthermore, the observed porosity and surface roughness of the material, as evidenced in the scanning electron microscopy (SEM) cross-sectional images (Figure 2), are key factors promoting the formation of these active sites and consequently contributing to the measured increase in the ECSA. The intricate surface morphology provides a larger interface for interaction with the analyte, facilitating improved charge transfer kinetics.

$$I_{p}=\pm 2.69\times 10^{5}\;n^{3/2}CD^{1/2}v^{1/2}A$$ 1

The ECSA is an important parameter for evaluating the effectiveness of the WE in electroanalysis. A larger ECSA is directly correlated with stronger electrochemical signals and more efficient electron transfer at the electrode surface. Importantly, determining the ECSA enables us to obtain the surface roughness factor (fr). This factor provides a quantitative measure of surface imperfections, which are directly linked to the electrode’s functional quality. Specifically, the roughness factor is the ratio between the ECSA and the electrode’s geometrically measured area (GSA), as described in eq 2:

$$\mathrm{fr}=\frac{\mathrm{ECSA}}{\mathrm{GSA}}$$ 2

The accurate determination of the electrochemically active surface area (ECSA) and subsequent calculation of the roughness factor are significant for understanding an electrode material’s intrinsic properties and optimizing its performance, including surface modification for specific electrochemical applications. According to the literature,^29–31^ a roughness factor approaching unity indicates a relatively smooth surface where the ECSA closely matches the geometric area. Conversely, larger deviations from unity signify increased surface roughness and imperfections. Here, the WE surface exhibited a roughness factor of approximately 1.80, which corroborates with the inherently irregular and nonplanar nature of the rGO/PAni composite (Figure 2). Additionally, this indicates a substantial increase in ECSA relative to its geometric counterpart, ensuring attractive features for further application in electroanalysis.

### 3.3 Voltammetric Study to Optimize the Experimental Conditions

Although the idea of using the electrochemical cells proposed in previous publications associated new extracting materials with electroanalysis, there is a need to advance studies to enable the simultaneous detection of a multiclass of pharmaceuticals as emerging environmental contaminants in water samples. Therefore, in this work, the construction of an alternative electrochemical cell for the detection of the drugs (acetaminophen, salicylic acid, and norfloxacin) was proposed. As such, the difference in the cell was the design in which the working electrode is fixed at the bottom. It should be noted that the synthesis of a thin film based on rGO/PAni to be used as a working electrode is highlighted due to its flexibility. Since this electrode has characteristics like a sheet of paper, it can be easily adapted to the bottom of the cell design. This allows the electrode system to be adjusted differently from conventional cells for the precise fit of a malleable, flexible, and easy-to-handle working electrode during the assembly and disassembly process after the analyses. Furthermore, the design of the electrochemical cell (details in Figure 1) allows, with the use of mechanical stirring of the solution, one to efficiently renew the electrode surface and minimize the effects of adsorption from the electrogenerated products.

As depicted in Figure 3, in the absence of the analytes, no oxidation peaks were observed within the scanned potential window, confirming the electrochemical inertness of the electrode under this potential window. Conversely, the introduction of each analyte individually (at a concentration of 100 μmol L^–1^) resulted in well-defined oxidation peaks. Specifically, acetaminophen (ACP) exhibited a single oxidation peak at a potential of 0.67 V vs Ag|AgCl|KCl_sat_ (against 0.74 V for simultaneous detection), while salicylic acid (SA) displayed an oxidation peak at 1.21 V vs Ag|AgCl|KCl_sat_ (against 1.23 V for simultaneous detection). For norfloxacin (NOR), two distinct oxidation peaks were observed at potentials of 0.83 and 1.15 V vs Ag|AgCl|KCl_sat_ (against 0.94 and 1.25 V for simultaneous detection), consistent with its known electrochemical oxidation pathway. Notably, under these initial conditions (without the use of a surfactant), the higher potential oxidation peak of NOR partially overlapped with the oxidation peaks of ACP and SA, respectively.

**Figure 3:**
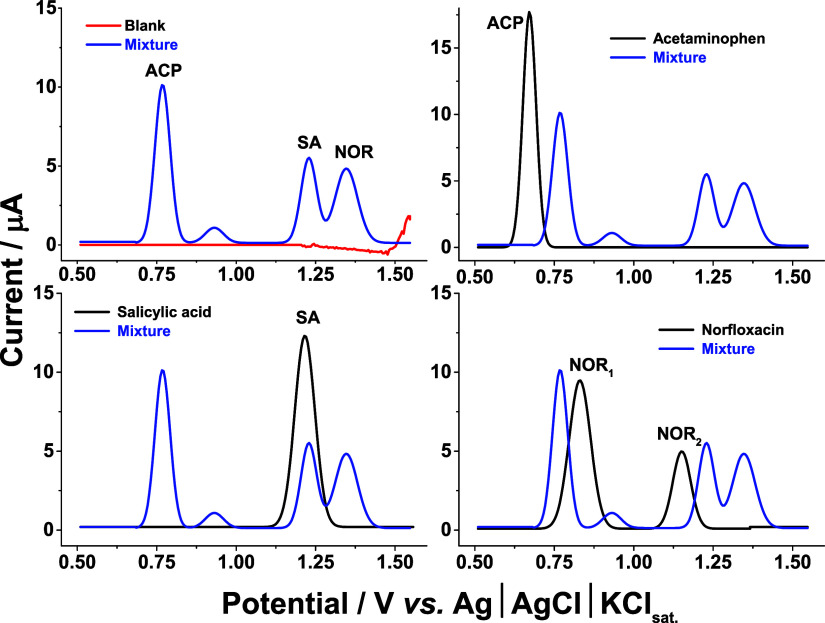
Baseline-corrected square-wave voltammograms on the rGO/PAni electrode recorded in the absence of the supporting electrolyte solution (sulfuric acid at 0.50 mol L^–1^) and presence of the analytes for the simultaneous detection of ACP, SA, and NOR at a concentration of 100 μmol L^–1^. Optimized conditions: step potential = 5.0 mV, pulse potential = 20 mV, and frequency = 30 Hz.

From Figure 3, the decreased peak current and shifting of the peak potential for NOR—when co-detected with ACP and SA—indicate interference in simultaneous multicomponent electroanalysis, a common occurrence in multicomponent voltammetry.^22,32,33^ Previous studies on mixed phenolics similarly observed shifts in oxidation peak potential and current for each component compared to single-analyte electroanalysis.^34^ This effect primarily results from the competitive adsorption of the multiple analytes on the active sites of the rGO/PAni electrode surface during simultaneous electroanalysis. To address this issue and enhance the resolution of the voltammetric signals for simultaneous determination, subsequent investigations focused on the optimization of both experimental parameters (e.g., supporting electrolyte composition, pH, and surfactants) and instrumental parameters (e.g., frequency, pulse potential of the square wave, and step potential). The potential implementation of surfactants as a strategy to improve peak separation through the modification of the electrode surface or analyte interactions was considered for future studies.

The incorporation of surfactants in electrochemical analyses is a crucial strategy to mitigate the detrimental effects of analyte and/or electrogenerated products adsorbed on solid electrode surfaces. Such adsorption phenomena can lead to electrode fouling and a consequent decrease in the electroanalytical performance. Surfactants are known to influence key voltammetric parameters, including peak potential and peak current.^35,36^ Furthermore, their application can minimize irreversible product adsorption, enhance the kinetics of electron transfer, and reduce the overpotential required for electrochemical reactions. Based on the successful utilization of neutral surfactants in studies involving structurally similar analytes,^37,38^ this investigation explored the impact of Triton X-100 and Tween 20 on the electrochemical behavior of the target compounds.

Figure 4 illustrates a significant shift in the peak potential upon the addition of these surfactants. Specifically, Triton X-100 induced a positive shift, while Tween 20 resulted in a negative shift, likely due to distinct surfactant–electrode interactions. Although the positive potential shift observed with both surfactants approached the supporting electrolyte discharge potential, they facilitated an improved separation of the oxidation peaks. This suggests a potential reduction in analyte adsorption on the rGO/PAni electrode surface mediated by surfactant–analyte interactions, creating more favorable conditions for the simultaneous determination of target analytes. Considering the enhancement in the peak current intensity for SA and NOR, coupled with the beneficial selectivity potential, Tween 20 was selected as the optimal surfactant for this study and used at a concentration of 50.0 μmol L^–1^. This choice aims to minimize adsorption on the rough rGO/PAni electrode surface and improve the selectivity of simultaneous electrochemical determination of target analytes.

**Figure 4:**
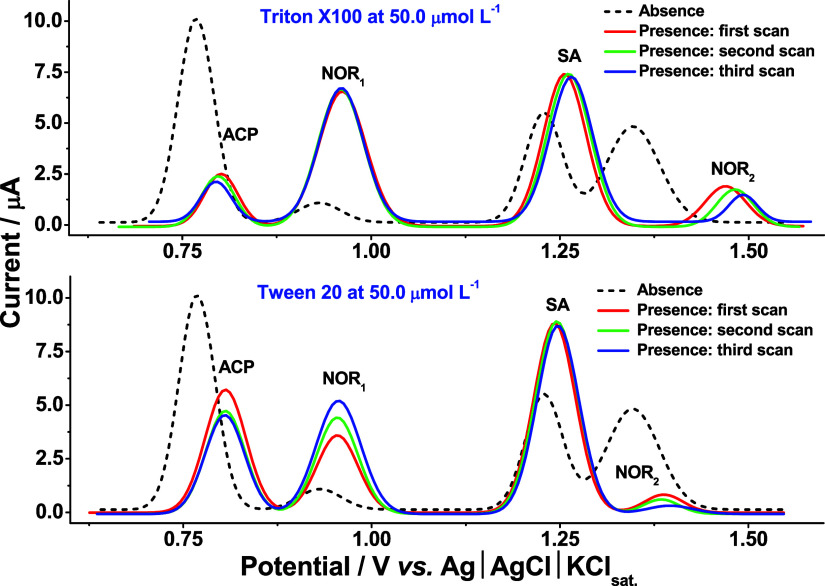
Baseline-corrected square-wave voltammograms on the rGO/PAni electrode recorded in the absence and presence of surfactants (Triton X-100 and Tween 20, both at a concentration of 50.0 μmol L^–1^) added in the supporting electrolyte solution (sulfuric acid at 50.0 mol L^–1^). Simultaneous detection of ACP, SA, and NOR was performed at a concentration of 100 μmol L^–1^. Optimized conditions: step potential = 5.0 mV, pulse potential = 20 mV, and frequency = 30 Hz.

The electrochemical performance of the rGO/PAni electrode and the electroactivity of organic analytes are critically dependent on the supporting electrolyte and its pH. As demonstrated by Martins et al.,^15^ rGO/PAni electrodes exhibit lower capacitance and consequently diminished performance in neutral and basic media compared to acidic medium, directly impacting their energy storage capabilities. This limitation extends to their function as working electrodes, hindering the oxidation of organic analytes under neutral or basic conditions. For instance, in a 40.0 mmol L^–1^ Britton–Robinson buffer at pH 5.0 (Figure S3A), only salicylic acid displayed electroactivity within the investigated potential window. Upon lowering the pH to 3.0 (Figure S3B) and 2.0 (Figure S3C), oxidation peaks corresponding to the oxidation of all three analytes became apparent. This highlights the crucial role of proton availability in their electrochemical oxidation, a significant consideration for electrochemical sensor development.

Furthermore, the choice of supporting electrolyte significantly influences the electrochemical processes. While lowering the pH in the B–R buffer enabled analyte oxidation, a 50.0 mmol L^–1^ sulfuric acid solution (Figure S3D) yielded a more well-defined voltammogram with enhanced peak current intensity compared to the 40.0 mmol L^–1^ B–R buffer across different pH values (Figure S3A–C). This suggests that, beyond pH, the higher ionic strength and specific ionic composition of the sulfuric acid solution create more favorable conditions for charge transfer and analyte oxidation in this system. In summary, these findings underscore the critical relationship among the supporting electrolyte, pH, and the electrochemical behavior of both electrode materials and analytes. Optimal performance in electrochemical devices and analytical methods necessitates a comprehensive understanding and careful selection of both the supporting electrolyte and the operating pH. The presented experimental data (Figures S3, SM) provide robust evidence for these conclusions.

### 3.4 Cleaning the rGO/PAni Electrode Surface by Mechanical Stirring

A critical requirement for working electrodes in electroanalysis is minimal susceptibility to adsorptive fouling by sample contaminants or electrogenerated products, alongside a wide working potential window. The conventional bottom-mounted working electrode configuration, coupled with a lab-made mechanical stirring solution, enables efficient surface renewal, mitigating the impact of electrogenerated product adsorption. However, for materials with high surface roughness such as rGO/PAni, surface fouling remains a concern.

Figure 5 illustrates the necessity of electrode surface cleaning for reproducible analytical responses. An initial voltammogram (Figure 5a) was followed by two successive measurements without intermediate cleaning (Figure 5b,c), revealing a significant decrease in peak current intensity, particularly for ACP and NOR. This decrease is attributed to the formation of a passivating film of inactive generated products on the rough rGO/PAni surface, hindering electrode activity and compromising its functionality. The effect of this adsorptive fouling intensified with subsequent scans. Notably, stirring the electrolyte solution for 2 min restored the peak current intensity to levels comparable to the initial measurement (Figure 5d). This demonstrates that mechanical agitation effectively cleans the electrode surface by removing adsorbed and/or electrogenerated products, thereby reactivating its electrochemical activity. Based on prior literature,^6,7^ a standardized cleaning protocol involving 2 min of solution agitation followed by a 2 min rest period was implemented before each electrochemical measurement to ensure consistent and reproducible results. This protocol addresses the inherent challenges associated with surface fouling on high-surface-area electrode materials.

**Figure 5:**
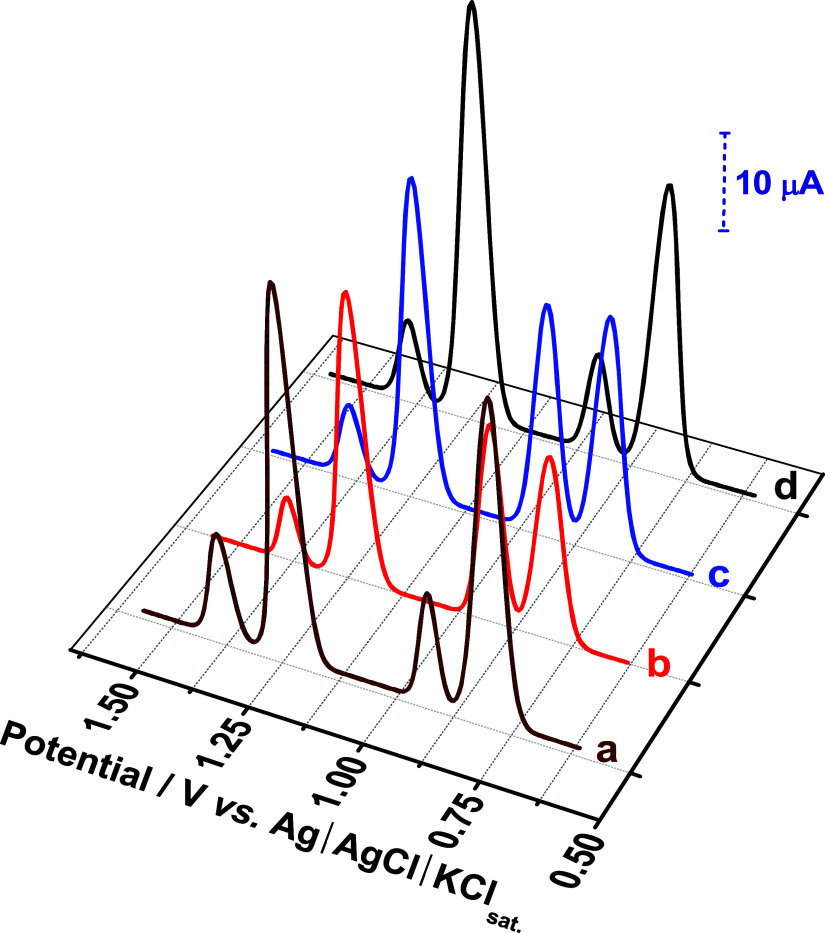
Baseline-corrected square-wave voltammograms on the rGO/PAni electrode recorded for the simultaneous detection of ACP, SA, and NOR at a concentration of 100 μmol L^–1^. The supporting electrolyte solution was sulfuric acid at 50.0 mol L^–1^ in the presence of surfactant Tween 20 at a concentration of 50.0 μmol L^–1^. (a) First measurement, (b, c) successive measurements without cleaning the electrode, and (d) measurement after stirring the solution to clean the rGO/PAni electrode surface. Optimized conditions: step potential = 5.0 mV, pulse potential = 20 mV, and frequency = 30 Hz.

To assess the impact of surface contamination on the rGO/PAni WE and the reproducibility of measurements, 15 successive square-wave voltammograms were recorded with a standardized cleaning procedure (2 min stirring, 2 min rest) between each scan. The resulting voltammograms, at specific intervals (Figure 6, voltammograms a–d), showed that while the fifth measurement (Figure 6, voltammogram b) has a minimal deviation, a noticeable decrease in peak current intensity for ACP and NOR was observed after the 10th measurement (Figure 6, voltammogram c). By the 15th measurement (Figure 6, voltammogram d), the characteristic oxidation peaks for ACP (*E*_p_ = 0.75 V) and NOR (*E*_p_ = 1.33 V) were no longer discernible, indicating a significant loss of electrode activity due to the rGO/PAni electrode surface fouling. This progressive decrease in signal intensity over successive measurements highlights the limitations of the cleaning protocol in fully mitigating contaminant adsorption and maintaining long-term electrode performance for rGO/PAni under these experimental conditions. Hence, to mitigate the effects of electrode fouling, which can occur due to the accumulation of electrogenerated products or interferents on the electrode surface, mechanical stirring of the solution was effectively used as an *in situ* cleaning step between each set of measurements. This stirring action aids in the removal of adsorbed species, and a surface renovation time of 2 min was chosen, as demonstrated in Figure 6. Accordingly, to ensure accurate data acquisition for requiring long-term electroanalysis, we used a new rGO/PAni electrode once this noticeable decline in signal intensity is observed.

**Figure 6:**
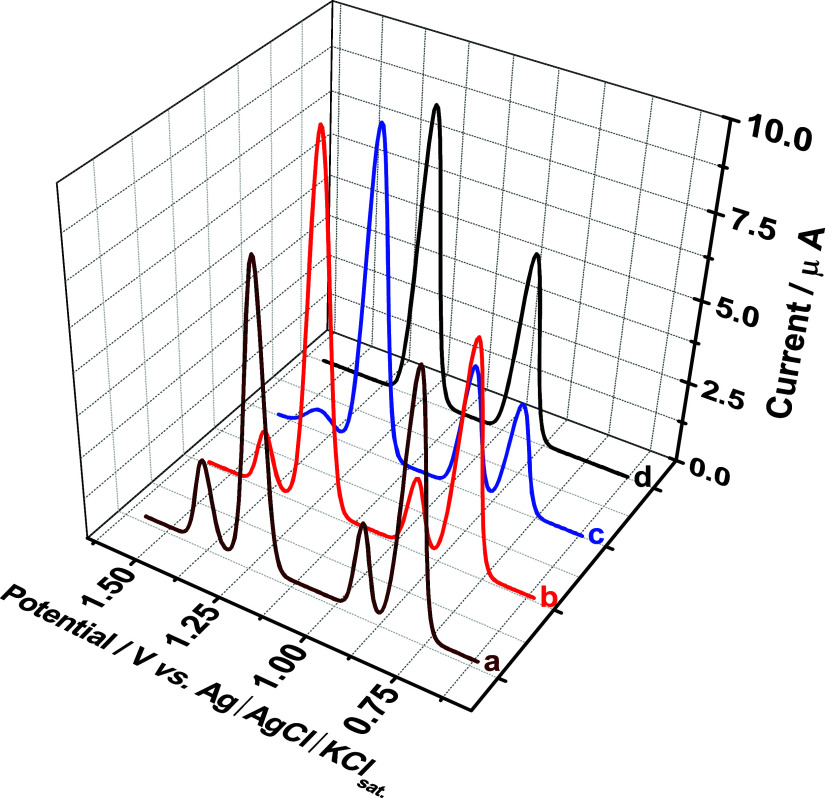
Baseline-corrected square-wave voltammograms on the rGO/PAni electrode recorded for the simultaneous detection of ACP, SA, and NOR at a concentration of 100 μmol L^–1^ after 15 measurements with 2 min intervals between each. (a) First measurement, (b) fifth measurement, (c) 10th measurement, and (d) 15th measurement. The supporting electrolyte solution was sulfuric acid at 50.0 mol L^–1^ in the presence of surfactant Tween 20 at a concentration of 50.0 μmol L^–1^. Optimized conditions: step potential = 5.0 mV, pulse potential = 20 mV, and frequency = 30 Hz.

Given that the proposed rGO/PAni WEs are manually fabricated and require periodic replacement due to inherent variability in the fabrication process, we conducted repeatability, reproducibility, and stability studies to assess the variations among different rGO/PAni electrodes (named rGO/PAni electrode 01, 02, and 03; Figure 7). Notably, as evidenced by the comparable voltammetric responses (Figure 7), independently prepared rGO/PAni WEs consistently deliver reliable simultaneous detection of ACP, SA, and NOR across different batches. Thus, acceptable variation in peak potential and peak current intensities effectively mitigates concerns related to manual fabrication variability and the required periodic electrode replacement.

**Figure 7:**
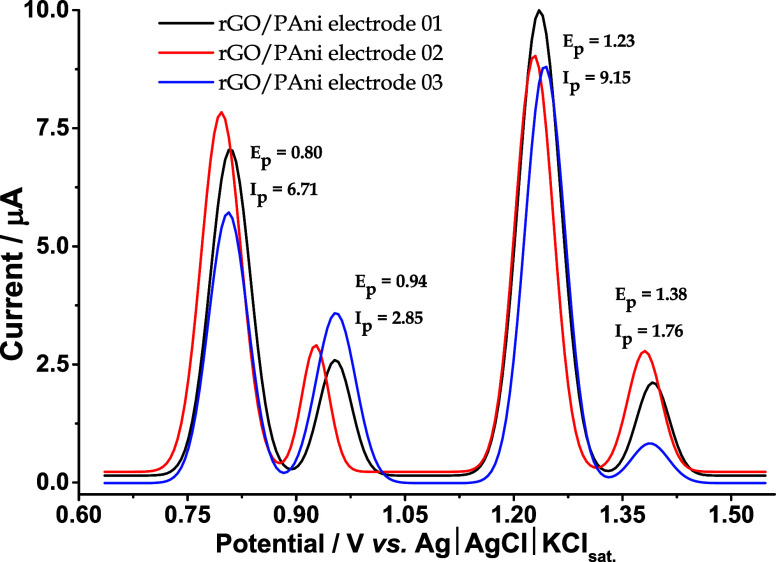
Baseline-corrected square-wave voltammograms were recorded to demonstrate the performance between different rGO/PAni electrodes for the simultaneous detection of ACP, SA, and NOR at 100 μmol L^–1^ each. Measurements were performed in 50.0 mol L^–1^ of sulfuric acid as the supporting electrolyte and 50.0 μmol L^–1^ of Tween 20 as the surfactant under optimized conditions: step potential = 5.0 mV, pulse potential = 20 mV, and frequency = 30 Hz.

In addition, the consistent and highly electrochemical activity across different batches is a direct result of the intrinsic, synergistic interaction between rGO and PAni, as detailed in our previous work.^15^ As demonstrated, PAni plays a vital role in binding graphene oxide sheets, which is essential for obtaining continuous, mechanically stable free-standing films. In the absence of the conductive polymer, the material becomes discontinuous and cannot function as a free-standing electrode. This binding effect arises from the π–π interactions between rGO and PAni, which promote strong interfacial adhesion within the nanocomposite. Moreover, our earlier study^15^ showed that the carboxylic groups present in GO act as dopants for PAni, converting it from its nonconductive emeraldine base form to the conductive emeraldine salt form. Together, the π–π stacking interactions and the doping mechanism synergistically increase electrical conductivity, facilitate charge transfer, and improve mechanical properties of the composite enough for multiclass pharmaceutical detection.

### 3.5 Analytical Performance and Proof-of-Concept Application

First, the best instrumental and experimental parameters were studied (data not shown), and the optimized values were based on peak current intensity, voltammetric resolution (evaluated by peak width at half height), and adequate peak-to-peak potential separation. To evaluate the electroanalytical performance of the rGO/PAni electrode, experiments were conducted in both the absence and presence of a sample matrix effect. The rGO/PAni electrode was positioned at the bottom of the electrochemical cell containing the supporting electrolyte, and the target analytes (acetaminophen (ACP), salicylic acid (SA), and norfloxacin (NOR)) were sequentially introduced under controlled stirring using a custom-designed device (detailed in Figure 1). As illustrated in Figure S4 (SM), successive additions of ACP, SA, and NOR under the applied electrochemical conditions resulted in a linear increase in peak current. This observation indicates a sensitive response of the rGO/PAni electrode surface to the concentrations of these model pollutants, suggesting its suitability as a sensing material.

The linear increase in peak current observed upon successive additions of the target analytes (Figure S4, SM) clearly indicates a selective response of the rGO/PAni electrode surface to simultaneous determination. This selective coupling with the high coefficients of determination (*R* ^2^ > 0.98) obtained from the external calibration curves (Figure S4, SM) confirms the accuracy of the developed rGO/PAni-based WE for quantitative analysis. The calculated limits of detection (LODs), calculated according to eq 3, where “σD” is the standard deviation of the intercept and “*m*” is the slope of the calibration curve, were below 24.0 μmol L^–1^ for all three pollutant models (further details in Table S1, SM), underscoring the high sensitivity of the developed electrochemical sensor.

$$\mathrm{LODs}=\frac{3\times \sigma D}{m}$$ 3

As a proof-of-concept for real-world applicability, a groundwater sample was spiked at two concentration levels (low and high) of the target analytes. The recovery studies, summarized in Table 1, demonstrated acceptable accuracy between the claimed and found concentrations with recovery values exceeding 74.3% for all spiked analytes. The successful simultaneous detection of ACP, SA, and NOR in a single measurement (Figure S4, SM) represents a significant advancement in electroanalytical techniques when comparing its performance with the HPLC method (Table 1) and previous work that involves preconcentration to reach accurate measurement at a low level of concentration.^6,7,32^ The well-defined and distinguishable electrochemical signals obtained for each analyte suggest minimal interference between them at the electrode surface under the optimized experimental conditions. This successful application of the flexible rGO/PAni electrode within the developed electrochemical cell provides further evidence of the viability of this electroanalytical approach. This suggests that the developed electrochemical cell and sensor configuration are robust to simultaneously detect the presence of multiple classes of pharmaceutical contaminants in a single measurement, which is enough to handle real-world sample analysis with minimal matrix effects.

**Table 1:** Addition–Recovery Experiments Carried Out to Evaluate the Quality of the Data Obtained in the Selective Determination of Multiclass-Based Pharmaceuticals Acetaminophen (ACP), Salicylic Acid (SA), and Norfloxacin (NOR) in Groundwater Samples

	proposed method			HPLC method		
analyte	added/μmol L^–1^	found ± μ^a^	recovery (%)	added/μmol L^–1^	found ± μ	recovery (%)
ACP	30.0	30.7 ± 3.8	102			
	60.0	44.6 ± 11	74.3	60.0	63.6 ± 0.20	106
SA	30.0	28.3 ± 2.1	94.4			
	60.0	66.3 ± 2.4	111	60.0	60.0 ± 0.20	100
NOR	40.0	34.0 ± 4.6	85.0			
	60.0	65.2 ± 6.7	109	90.0	90.1 ± 0.15	100

a μ: confidence interval for three determinations at a 95% of confidence level.

The promising results achieved can be attributed to a strategic approach that addressed the inherent limitations in conventional electrochemical cell designs. Specifically, the integration of a flexible rGO/PAni electrode within a carefully optimized cell architecture proved to be crucial in enhancing the analytical performance. With this new design, it was possible to simultaneously determine three analytes (included in the list of emerging contaminants) without the need for a previous separation step. This approach effectively addresses limitations often associated with electrochemical sensors, such as electrode fouling and the challenge of simultaneous multianalyte detection, paving the way for more robust and practical electrochemical solutions for environmental analysis. Finally, the system demonstrates sensitivity and selectivity, making it highly suitable for use in real samples, where precise analyte measurement is essential. The electrode design enhances analyte access and ensures consistent and reliable analytical performance, matching or surpassing the effectiveness of previously described bare or modified electrodes for detecting individual analytes (Table S2, SM).

## 4 Conclusions

We have demonstrated a lab-made electroanalytical system featuring a flexible rGO/PAni electrode strategically integrated into a bottom-mounted electrochemical cell. This design capitalizes on the synergistic properties of rGO/PAni—namely, its high conductivity, expansive surface area, and robust electroactivity—to achieve a selective WE capable of simultaneous multianalyte detection of pharmaceuticals in aqueous samples. Beyond its electroanalytical performance, the device offers significant practical advantages: low fabrication cost, straightforward operation, minimal measurement steps, and inherent *in situ* electrode surface regeneration. These benefits underscore its promise to reduce costs, simplify handling, and minimize the number of steps required for pharmaceutical contamination monitoring. Future research could focus on further optimizing the electrode material and cell design, investigating the system’s performance in a wider range of environmental matrices, and exploring its long-term stability and applicability for *in situ* monitoring of pharmaceutical contamination.

## Supplementary Material



## Notes

Additional experimental details, including tables and figures, showing the rGO/PAni electrode characterization, experimental parameter optimization, figure of merits, as well as the comparative analytical performance of the rGO/PAni electrode (PDF)
