# Supplementary material for: Lab-Made Electrochemical System with Flexible rGO/PAni Electrode for Selective Multiclass Pharmaceutical Detection
Source: ACS Omega. 2026 Jan 21;11(4):6591–601. doi: 10.1021/acsomega.5c11695 (PMC12878775; doi:10.1021/acsomega.5c11695)
Supplement: Supplementary file 1 [file ao5c11695_si_001.pdf]

Supplementary Information

Lab-made electrochemical system with flexible rGO/PAni electrode for selective multiclass pharmaceutical detection

Layne Taynara Santos Zanon[1], Vitor Hugo Neto Martins[1], Liriana Mara Roveda[1], Luis Gustavo do Espírito Santo Mendes[1], Claudio Teodoro de Carvalho[1], Raphael Rodrigues[1], Victor Hugo Rodrigues de Souza[1], Magno Aparecido Gonçalves Trindade[1,*]

[1] Faculdade de Ciências Exatas e Tecnologia, Universidade Federal da Grande Dourados, Rodovia Dourados-Itahum, km 12. Dourados-MS, 79804-970, Brazil

*To whom correspondence should be addressed:

Magno A. G. Trindade

E-mail: magnotr@gmail.com and magnotrindade@ufgd.edu.br

Phone: +55 67 3410-2092

Fax: +55 67 3410-2072

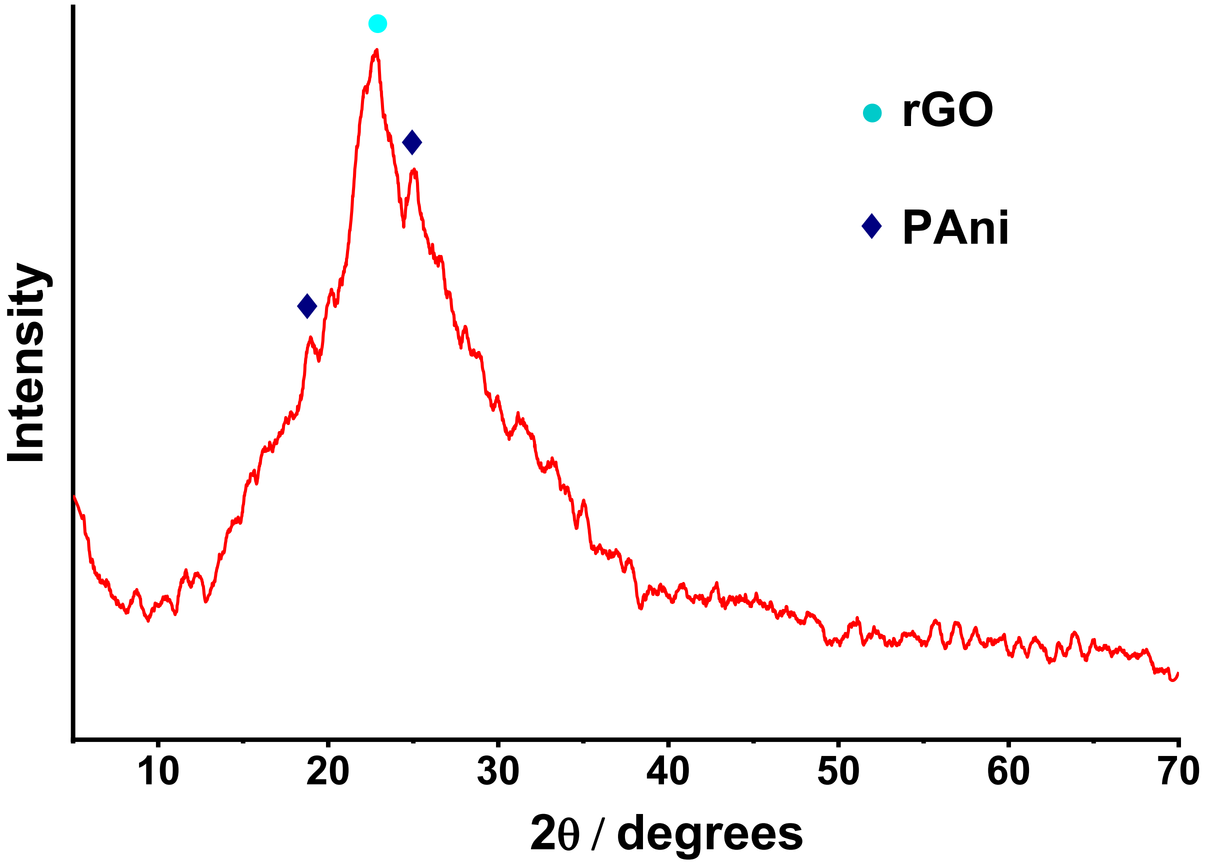

Figure S1. X-ray diffraction analysis (XRD) pattern for the rGO/PAni modified electrode.

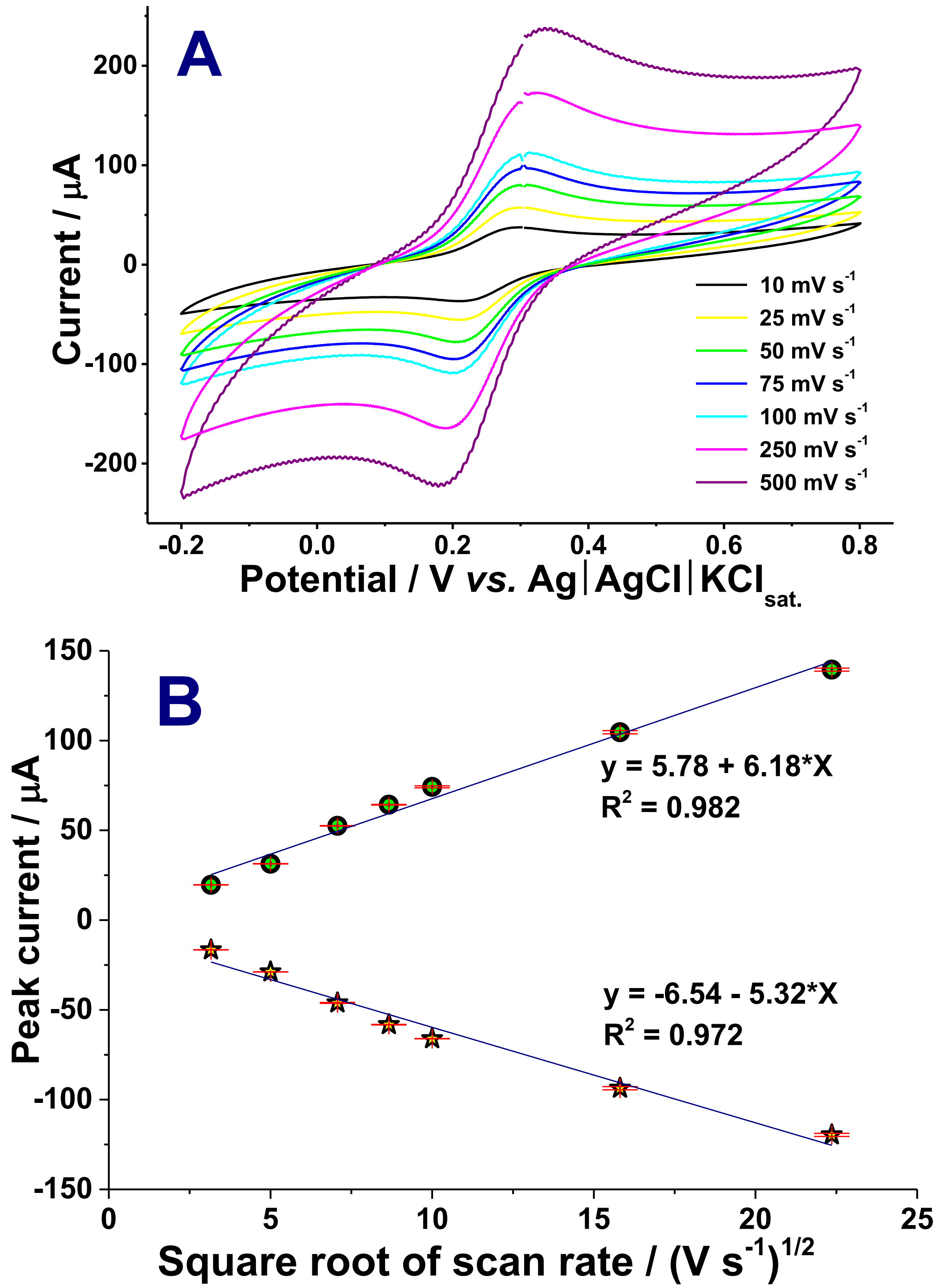

Figure S2. (**A**) Cyclic staircase voltammograms recorded for the rGO/PAni at different scan rates in the presence of the redox probe ($[Fe(CN)_6]^{4-}$/ $[Fe(CN)_6]^{3-}$) (1.00 mmol L^{-1}) in KCl (50.0 mmol L^{-1}). (**B**) The corresponding linear plot shows the redox cathodic and anodic peak currents versus square root of scan rate.

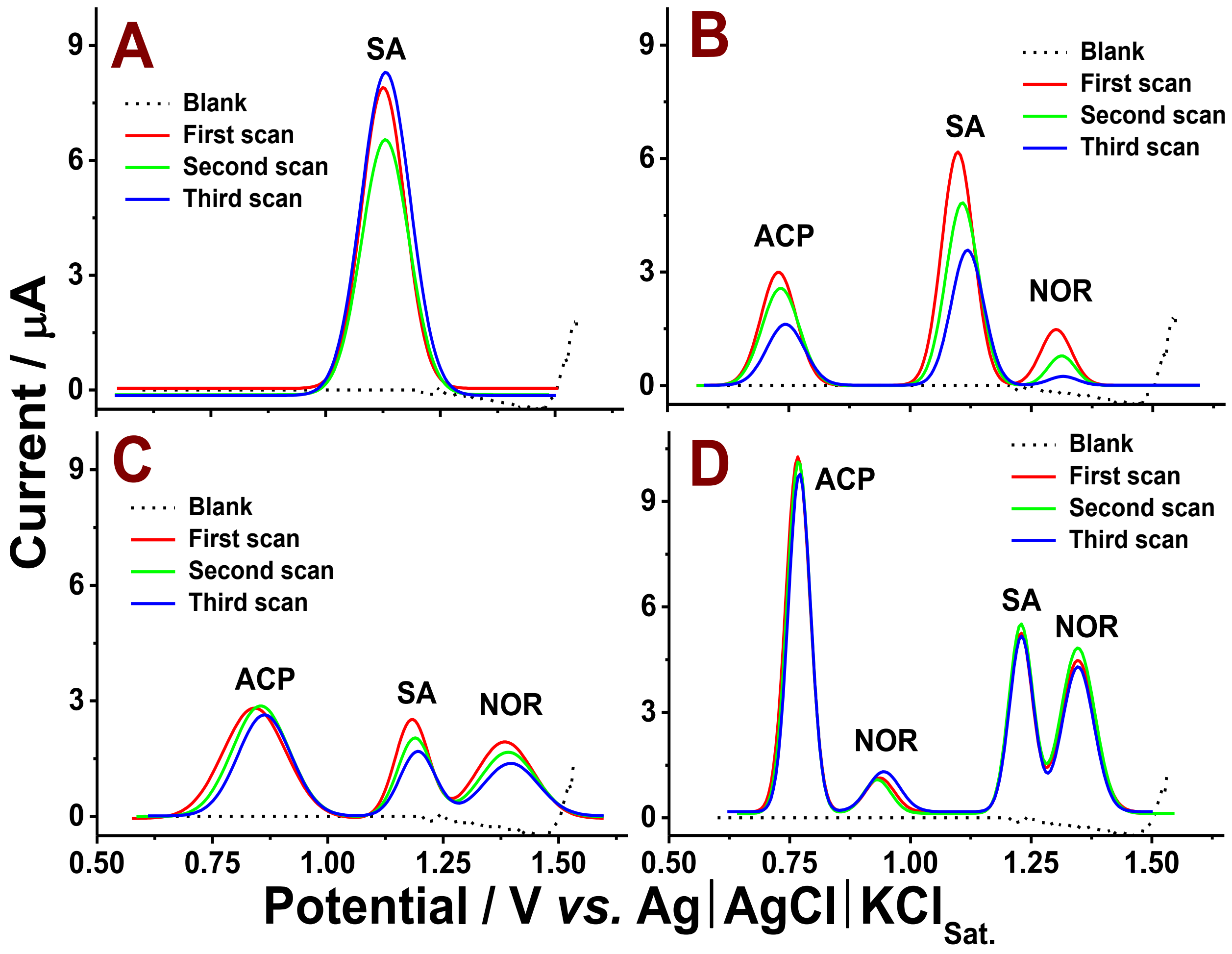

Figure S3. Baseline-corrected square-wave voltammograms on the rGO/PAni electrode recorded for the simultaneous detection of acetaminophen (ACP), salicylic acid (SA), and norfloxacin (NOR) at a concentration of 100 μmol L^{-1}. The supporting electrolyte solution was: (**A**) B-R buffer (40.0 mmol L^{-1}) at pH 5.0 with the addition of KCl (0.50 mol L^{-1}) to adjust the ionic strength; (**B**) B-R buffer (40.0 mmol L^{-1}) at pH 3.0 with the addition of KCl (0.50 mol L^{-1}) to adjust the ionic strength; (**C**) only in B-R buffer (40.0 mmol L^{-1}) at pH 2.0 and (**D**) only in sulfuric acid at 0.50 mol L^{-1}. Optimized conditions: step potential = 5.0 mV, pulse potential = 20 mV, and frequency = 30 Hz.

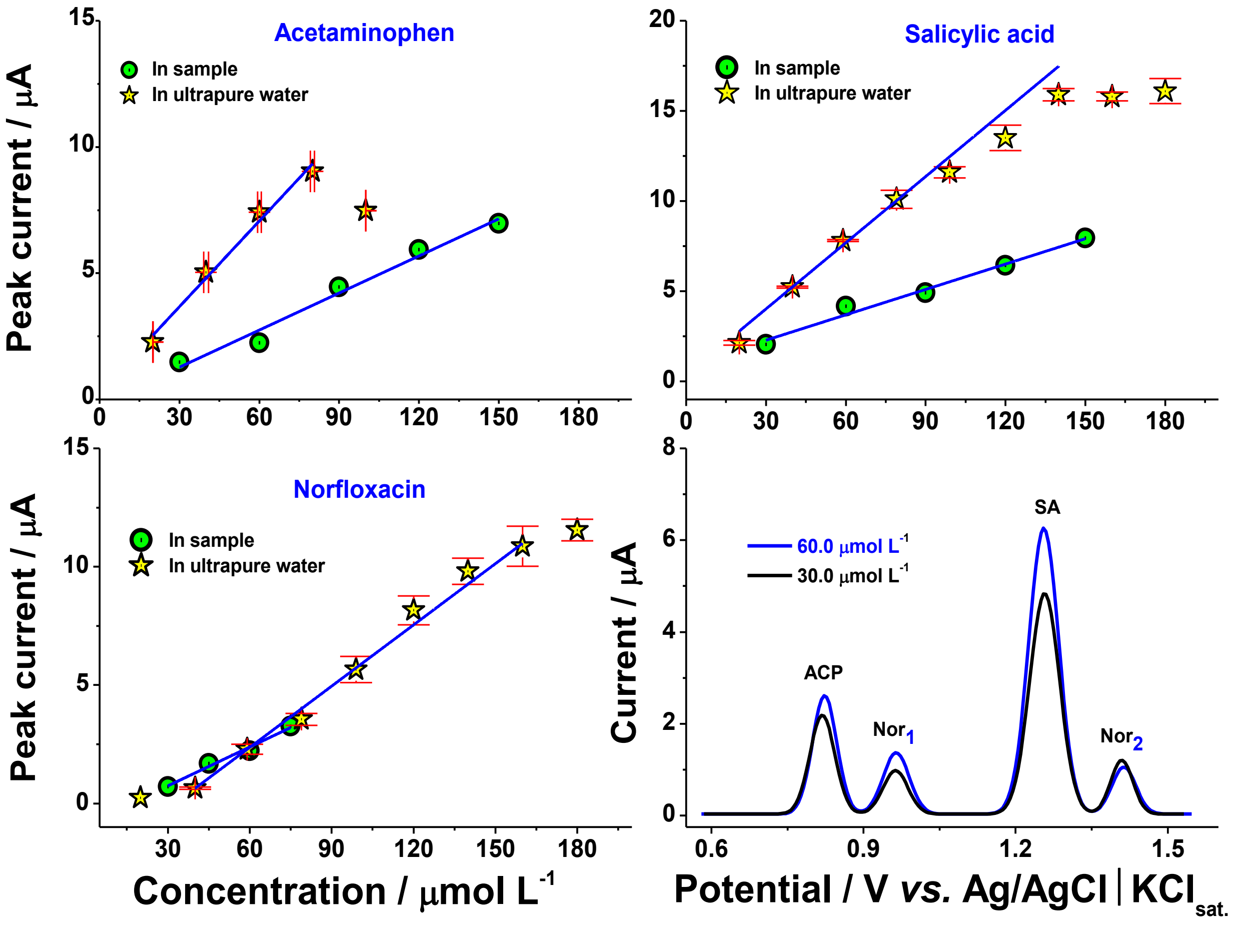

Figure S4. Analytical calibration for the dependence of oxidation peak currents on acetaminophen (ACP), salicylic acid (SA), and norfloxacin (NOR) concentrations. Square-wave voltammogram recorded using rGO/PAni-based working electrode for detecting target analytes in groundwater sample spiked at 30.0 and 60.0 μmol L^{-1}. The supporting electrolyte solution was sulfuric acid (0.50 mol L^{-1}) prepared in the groundwater sample. Optimized conditions: pulse potential = 10 mV, step potential = 5.0 mV, and frequency = 30 Hz.

Table S1. Analytical parameters obtained from calibration curves, comparing the performance of the rGO/PAni-based working electrode in detecting acetaminophen (ACP), salicylic acid (SA) and norfloxacin (NOR) in ultrapure and groundwater samples.

Analyte	Condition	Linear range (μmol L^{-1})	Linear equation	R^2	LOD (μmol L^{-1})
**ACP**	Ultrapure water	20.0 – 80.0	Y = 0.28 + 0.11*X	0.994	6.65
	Groundwater	30.0 – 150	Y = -0.19 + 0.049*X	0.990	9.10
**SA**	Ultrapure water	20.0 – 140	Y = 0.35 + 0.12*X	0.988	7.50
	Groundwater	30.0 – 150	Y = 0.99 + 0.047*X	0.992	8.75
**NOR**	Ultrapure water	20.0 – 100	Y = -2.8 + 0.086*X	0.997	5.22
	Groundwater	30.0 – 75.0	Y = -0.90 + 0.055*X	0.993	10.5

Table S2. Comparative performance of the proposed rGO/PAni WE with other modified and bare WEs for individual or simultaneous determination of acetaminophen (ACP), salicylic acid (SA) and norfloxacin (NOR) in different samples.

Electrode material	Analyte	Linear range (µmol L^{-1})	LOD (µmol L^{-1})	Ref.
GCE/CoTATPAPc	ACP	0.020 – 0.36	0.0063	[1]
GCE/poly(CoTAPOPMPPc)	SA	5.00 – 120	2.00	[2]
GCE/KB-poly(CoTAPOPMPPc)	SA	5.00 – 120	1.50	[2]
CB-PLA	AAP	0.500 – 23.0	0.210	[3]
3D-printed chip	ACP	100 - 300	20.0	[4]
	SA	100 – 300	3.00	
TiO2/PB/AuNPs/CMK-3/Nafion sensor	ACP	10.0 – 52.0	0.210	[5]
NiONPs-GO-CTS: EPH/GCE	ACP	0.10 – 2.90	6.70	[6]
C-HAP modified GCE	ACP	0.010 – 1310	0.140	[7]
NNC-PPY/SWCNTs/SPE sensor	ACP	0.050 – 40.0	0.072	[8]
Au–Ag-ANCCs decorated with f-MWCNT-CPE	NOR	0.00090 - 200	0.00014	[9]
BDD	NOR	1.50 – 5.50	0.520	[10]

LOD: Limit of detection

References

[1] Palanna, M., Mohammed, I., Aralekallu, S., Nemakal, M., Sannegowda, L. K. Simultaneous detection of paracetamol and 4-aminophenol at nanomolar levels using biocompatible cysteine-substituted phthalocyanine. New Journal of Chemistry 2020; 44: 1294–306.

[2] Giddaerappa, N. M., Mohammed I., Sannegowda, L. K. Mannich reaction derived phthalocyanine polymer for electrochemical detection of salicylic acid. Inorganica Chim Acta 2020; 512: 119895. https://doi.org/https://doi.org/10.1016/j.ica.2020.119895.

[3] Roveda, L. M. *et. al*. Fully 3D printed electrochemical cell design with integrated electrodes array: A simple and versatile tool for sustainable electroanalysis. Sustainable Materials and Technologies 2025; 43: e01325.

[4] Gonçalves, D. A., Estadulho, G. L. D., Guima, K.-E., Martins, C. A. Multi-electrode platform for selective electrochemical sensing: 3D-printed insulating plastic is turned into a five-electrodes chip. Talanta 2022; 250: 123705.

[5] Pollap, A., Baran, K., Kuszewska, N., Kochana, J. Electrochemical sensing of ciprofloxacin and paracetamol in environmental water using titanium sol based sensor. Journal of Electroanalytical Chemistry 2020; 878: 114574.

[6] Santos, A. M., Wong, A., Almeida, A. A., Fatibello-Filho, O. Simultaneous determination of paracetamol and ciprofloxacin in biological fluid samples using a glassy carbon electrode modified with graphene oxide and nickel oxide nanoparticles. Talanta 2017; 174: 610-618.

[7] Anitta, S., Sekar, C. Voltammetric determination of paracetamol and ciprofloxacin in the presence of vitamin C using cuttlefish bone-derived hydroxyapatite sub-microparticles as electrode material. Results in Chemistry 2023; 5: 100816.

[8] Shalauddin, Md., Akhter, S., Basirun, W. J., Lee, V. S., Johan, M. R. A metal free nanosensor based on nanocellulose-polypyrrole matrix and single-walled carbon nanotube: Experimental study and electroanalytical application for determination of paracetamol and ciprofloxacin. Environmental Nanotechnology, Monitoring & Management 2022; 18: 100691.

[9] Adane, W. D., Chandravanshi, B. S., Getachew, N., Tessema, M. A. Cutting-Edge Electrochemical Sensing Platform for the Simultaneous Determination of the Residues of Antimicrobial Drugs, Rifampicin and Norfloxacin, in Water Samples. Analytica Chimica Acta 2024; 1312: 342746.

[10] Rosa, T. M, Roveda, A. C., da Silva, G. W. P., Martins C. A., Oliveira, P. R., Trindade, M. A. G. Electrochemical cell designed for in situ integrate microextraction and electroanalysis: Trace-level determination of norfloxacin in aqueous samples. Talanta 2019;196.
